# Viral Particle-Mediated SAMHD1 Depletion Sensitizes Refractory Glioblastoma to DNA-Damaging Therapeutics by Impairing Homologous Recombination

**DOI:** 10.3390/cancers14184490

**Published:** 2022-09-16

**Authors:** Waaqo Daddacha, Dominique Monroe, Kristen Carver, Edidiong R. Usoro, Ahmet Alptekin, Hongyan Xu, Satoru Osuka, Ali S. Arbab, Daitoku Sakamuro

**Affiliations:** 1 Department of Biochemistry and Molecular Biology, Medical College of Georgia, Augusta University, Augusta, GA 30912, USA; 2 Georgia Cancer Center, Augusta University, Augusta, GA 30912, USA; 3 Department of Population Health Sciences, Medical College of Georgia, Augusta University, Augusta, GA 30912, USA; 4 Department of Neurosurgery, Heersink School of Medicine, The University of Alabama, Birmingham, AL 35233, USA

**Keywords:** SAMHD1, DNA damage, DNA repair, TMZ, radiation therapy, malignant glioma, glioblastoma, Vpx, homologous recombination, irradiation

## Abstract

**Simple Summary:**

Glioblastoma (GBM) is a lethal and common primary brain tumor that accounts for about 50% of all diagnosed malignant gliomas. Despite aggressive standard-of-care treatment of surgical resection followed by γ-irradiation (IR) and DNA alkylating agent temozolomide (TMZ), the average post-diagnosis survival time for a GBM patient remains at 15 months. This is mainly due to acquired resistance and limited therapeutic options. Sterile alpha motif and HD domain-containing protein 1 (SAMHD1) supports DNA double-strand break repair by promoting homologous recombination (HR) and it can be targeted to proteasomal degradation by viral protein X (Vpx). We aim to evaluate whether depleting SAMHD1 sensitizes refractory GBM to IR and TMZ, and the possibility of utilizing Vpx as therapeutic tool. We report that SAMHD1 is highly expressed in GBM. Vpx-mediated SAMHD1 depletion impaired HR and sensitized GBM cells to IR and TMZ. Our finding demonstrates the potential therapeutic benefit of targeting SAMHD1 with Vpx in GBM.

**Abstract:**

The current standard-of-care treatment for glioblastoma includes DNA damaging agents, γ-irradiation (IR) and temozolomide (TMZ). These treatments fail frequently and there is limited alternative strategy. Therefore, identifying a new therapeutic target is urgently needed to develop a strategy that improves the efficacy of the existing treatments. Here, we report that tumor samples from GBM patients express a high level of SAMHD1, emphasizing SAMHD1’s importance. The depletion of SAMHD1 using virus-like particles containing Vpx, VLP(+Vpx), sensitized two independent GBM cell lines (LN-229 and U-87) to veliparib, a well-established PARP inhibitor, and slowed cell growth in a dose-dependent manner. In the mouse GBM xenograft model, Vpx-mediated SAMHD1 depletion reduced tumor growth and SAMHD1 knockout (KO) improved survival. In combination with IR or TMZ, SAMHD1 KO and exposure to 50% growth inhibitory dose (gID50) of VLP(+Vpx) displayed a synergistic effect, resulting in impaired HR, and improved LN-229 cells’ sensitivity to TMZ and IR. In conclusion, our finding demonstrates that SAMHD1 promotes GBM resistance to treatment, and it is a plausible therapeutic target to improve the efficacy of TMZ and IR in GBM. Furthermore, we show that Vpx could be a potential therapeutic tool that can be utilized to deplete SAMHD1 in GBM.

## 1. Introduction

Glioblastoma (GBM) is a lethal and frequently diagnosed form of primary malignant central nervous system (CNS) tumors, accounting for about 50% of all diagnosed malignant gliomas [1,2]. GBM conventional treatment involves maximum surgical resection followed by radiation therapy (RT) and chemotherapy using genotoxic agents such as temozolomide (TMZ). Despite some success with these treatments, the prognosis of GBM patients has not shown notable improvement over the last several decades. Overall, the average post-diagnosis survival time for patients with GBM is approximately 15 months. [3,4]. In 2016, the WHO classified gliomas based on molecular parameters in addition to previous classification based on histological parameters. Under histologic parameters, gliomas are classified in to grades I to IV. While grade I represents the least aggressive glioma, grade IV represents the most aggressive glioma which corresponds to GBM. Under molecular parameters based on isocitrate dehydrogenase (IDH) mutation, 95% of primary GBM are associated with IDH wild-type (IDH WT) and low-grade glioma (LGG) with wild-type IDH are rare [5]. Furthermore, IDH WT GBM is resistant to therapy and displays extremely poor outcome [6].

Subsequent cancer cell death resulting from γ-irradiation (IR) and TMZ treatment involves induction of DNA damage [7,8]. However, GBM cells with intact or dysregulated DNA repair pathways may develop resistance to these treatments. It is well established that IR induces DNA double-strand break (DSB) and that inhibiting the homologous recombination (HR) pathway enhances IR efficacy in diverse cancer types, including GBM [9,10]. The efficiency of TMZ, an alkylating agent, partly depends on methylation of the O[6]-methylguanine-DNA methyltransferase (MGMT) promoter and the mismatch repair pathway [11]. However, there is overwhelming evidence that DSB repair plays a role in resistance to alkylating agents. Importantly, impaired HR can sensitize GBM cells to TMZ [12,13]. These findings demonstrate the potential benefit of targeting the HR pathway to improve the efficacy of TMZ and IR in refractory GBM.

Sterile alpha motif and HD domain-containing protein 1 (SAMHD1) was initially associated with Aicardi-Goutières Syndrome (AGS) and has been identified as a deoxyribonucleotide triphosphohydrolase (dNTPase) with a well-defined human immunodeficiency virus type one (HIV-1) restricting function [14,15]. SAMHD1 promotes resistance to dNTP analog chemotherapy [16,17], and its mutations have been linked to human cancers [18,19], exhibiting its relevance beyond viral infection and importance to cancer. Moreover, SAMHD1 promotes DNA processing at stalled replication forks [20] and supports genomic integrity by resolving R-loops [21], which is consistent with SAMHD1 accumulation at the DNA damage site [22]. We reported a unique role for SAMHD1 in promoting DNA-end resection to facilitate DSB repair through HR [23]. TMZ and IR therapeutic values partly rely on inducing DSB, and impaired DSB repair sensitizes GBM cells. Thus, SAMHD1 is a plausible target to enhance the efficacy of these conventional therapeutics in GBM.

This study utilized viral protein as a tool to selectively deplete SAMHD1. Human immunodeficiency virus type 2 (HIV-2) and simian immunodeficiency virus (SIV) accessory protein X (Vpx) interact with SAMHD1 and recruit the CRL4-DCAF1 E3 ubiquitin ligase complex, leading to SAMHD1 polyubiquitination and subsequent proteasomal degradation [24]. Successful delivery of Vpx into target cells can be achieved by packaging it in a virus-like particle (VLP), providing a vector-based system to transiently deplete cellular SAMHD1 [25]. Vectors derived from diverse viruses, including lentiviruses, are already being utilized to deliver a peptide, protein, or gene for medical applications such as vaccines and gene therapy (reviewed in [26,27]). Furthermore, because VLPs do not contain genomic material, they provide a safer yet effective system to protect and transiently deliver a protein of interest into target cells while leaving the host genome intact.

In GBM, the likelihood of targeting DNA damage pathways to develop novel therapies or to enhance the efficacy of currently available therapeutic options has been a research interest for decades. Some of such therapies have advanced to clinical trials [28,29]. However, there is still no solution to overcome the refractory nature of GBM, and patient outcomes remain extremely poor. This study demonstrates the value of utilizing VLP-mediated Vpx delivery to induce cellular SAMHD1 depletion to enhance the sensitivity of GBM cells to IR and TMZ.

## 2. Materials and Methods

***Cell Culture and transfection***. Glioma cell lines H4, LN-229, and U-87 were grown in DMEM (Gibco) supplemented with fetal bovine serum (FBS). Human astrocytes (HA1890) were a kind gift from Dr. Hedong Li’s laboratory and were grown in DMEM/F-12 GlutaMax (Gibco) supplemented with FBS, 2% B-27 (Gibco), and 3.5 mM (0.35%) of glucose solution (Gibco) (Base Media). Immediately prior to plating, 100 ng/mL bFGF (Gibco) and 10 ng/mL HB-EGF (Sigma-Aldrich) were added to prepare complete media. Cells were incubated at 37 °C and 5% CO_2_, and the medium was changed every three days. Astrocytes were grown to 80% confluence before being passaged. Transfection was performed according to the manufacturer’s instructions using Lipofectamine 3000 (Thermo Fisher Scientific) with a slight modification of 1:1 lipofectamine 3000 to p3000 reagent. Transfected cells were incubated at 37 °C in serum-free DMEM for 4 h and later replaced with FBS-supplemented DMEM. The cells were incubated at 37 °C, and the medium was changed every three days or until the experimental endpoint.

***Western blot***. The desired number of cells was harvested and lysed for 30 min on ice in BC200 buffer (200 mM NaCl, 25 nM Tris-Cl (pH 8), 0.2% Np-40, 10% glycerol, 1 mM DTT, and 1 mM EDTA (pH 8)), which was supplemented with protease inhibitors before each use. The protein concentration of each lysate was measured using the Bradford assay after centrifugation at the maximum speed for 10 min at 4°C. Total protein lysates (10–40 µg) were resolved on SDS gel and visualized with an LI-COR Odyssey observer after probing with primary antibodies followed by Alexa Fluor anti-mouse or anti-rabbit secondary antibodies (Life Technologies). The membranes were visualized using a Li-Core Odyssey system and analyzed.

***Generation of SAMHD1 knockout GBM cells***. The SAMHD1 KO cells were generated as described previously [23]. Briefly, LN-229 cells were seeded on a six-well plate at 2.5 × 10^5^ cells/well, allowed to adhere overnight, and transfected as described above with all-in-one CRISPR/Cas9-GFP vectors containing two different guide RNAs (Sigma Aldrich, St. Louis, MO, USA). The medium was changed 4 h after transfection, and cells were incubated for an additional two days before harvesting for single-cell sorting. Cells were resuspended in DMEM supplemented with FBS, and those expressing GFP were sorted in a 96-well plate (a single cell/well) using fluorescence-activated cell sorting (FACS). Sorted cells were grown until semiconfluent, transferred to 6-well plates, expanded, and harvested for validation of gene knockout by western blotting. Confirmed SAMHD1 knockout cells were utilized for downstream experiments or were frozen and stored for later use.

***Virus-Like Particle (VLP) Preparation***. VLPs containing the retrovirus accessory protein Vpx (Vpx) were produced as previously described [14,30], with minor modifications. Briefly, 293T cells were co-transfected with a transfection complex (40 µg PEI, 40 µg packaging plasmid (p(−Vpx) or p(+Vpx), and 20 µg PCMV-VSV-G at a 2:1 mass ratio.) Media were changed 24 h post-transfection, and media containing the VLPs were collected until the monolayer was disrupted. The VLPs were concentrated in the presence of a 25% sucrose cushion by centrifugation at 4 °C at 280,000 rpm for 90 min. The concentrated VLPs were resuspended in DMEM, and the particle quantity was assessed using a p24 ELISA kit.

***Ectopic Xenograft Model***. Female athymic nude mice were purchased from Charles River NCI at Fredrick at the age of 5 weeks. LN-229 cells were resuspended in a serum-free medium at a concentration of 5 × 10^6^ cells/mL. Cells (1 × 10^6^) were injected subcutaneously into either flank of each mouse. After approximately one month, when the tumors had a volume greater than 100 mm^3^, the left flanks were injected with 0.1 mL of VLP(+Vpx) for three consecutive days, then once a week. The tumor size was measured with a Kynup Digital Caliper (USQC03301915) and volume was calculated using the following formula: π/6 × length × width^2^.

***Mouse Brain Tumor Model***. Seventeen athymic nude mice were purchased from Charles River NCI at Fredrick at the age of 5 weeks and separated into two groups. One group for LN-229 SAMHD1 KO (8 mice) or LN-229 WT (9 mice) xenograft establishment. All mice were anesthetized with 100 mg/kg ketamine and 15 mg/kg xylazine in the intraperitoneal space. Prior to injection, each mouse was swabbed with betadine and ethanol solutions, and the eyes were coated with a Lacrilube to reduce dryness. Then, a 1 cm incision was made 2 mm right of the midline, starting 1 mm retro-orbitally. The skull was exposed, and a hole was drilled 2 mm right of the bregma while not piercing the dura. A #2701 10 µL Hamilton syringe with a #4 point, a 26-gauge needle containing 5 × 10^5^ LN-229 SAMHD1 KO or LN-229 WT cells in 3 µL media was lowered to a depth of 3.5 mm and then raised to a depth of 2.5 mm. The cells were injected gradually at a rate of 0.5 uL/min. After injection, the syringe was gradually removed to reduce reflux from the injection site. The exposed skull was coated with bone wax and swabbed with betadine before the skin was sutured. Buprenorphine (~0.05 mg/kg) was administered subcutaneously and monitored. Mice were sacrificed when more than 10% weight loss had occurred from the initial body weight before surgery or if the mouse showed signs of severe distress according to the approved protocol.

***Cell proliferation and Viability assay***. Cells were seeded in a 96-well plate at a density of 1 × 10^3^ cells/well in triplicate, allowed to adhere overnight, and transduced with VLPs containing Vpx or VLP with no Vpx. The medium was replaced 24 h post-transduction when the maximum Vpx mediated-SAMHD1 degradation efficiency was observed. Subsequently, the cells were treated with TMZ or IR and incubated for 96 h. Cell viability was then assessed by incubating cells with media containing 10% AlamarBlue reagent (Thermo Fisher DAL1100) at 37 °C. The fluorescence signal was measured at 540 nm excitation and 590 nm emission, and the viability fractions were normalized to vehicle-treated controls exposed to identical transduction or transfection conditions. For the proliferation assay, cells were seeded in 6-well plates (50,000 cells/well), harvested after 24, 48, 72, 96, and 120 h, diluted in trypan blue, and counted to assess proliferation.

***Immunofluorescence Assay***. LN-229 cells were seeded on coverslips, transduced with VLPs as described above, and irradiated. The cells were incubated for 6- and 24-h post irradiation, washed with PBS, fixed with 1% PFA, and treated with 0.5% Triton X-100 for permeabilization. The permeabilized cells were blocked with 5% BSA and probed with γH2AX and RPA70 primary antibodies, followed by Alexa Fluor 488 or 555 secondary antibodies. The coverslips were mounted with DAPI Fluoromount-G (Sothern Biotech) and analyzed. For each condition, 50 cells were counted based on DAPI staining as total cell count, γH2AX foci were used as an indicator of percent damage, and out of the γH2AX foci positive cells, RPA70 foci positive cells were used as percent end resection indicator.

***Clonogenicity Assay***. Cells were seeded at 200 cells/well in 6-well plates in triplicate and grown for two weeks or until the control reached a minimum of 50 cells/colony. The colonies were then fixed, stained with crystal violet, and allowed to dry. Colonies with 50 or more cells were counted, and the results were plotted.

***Quantification of cellular dNTP pools***. Cellular dNTP was extracted as described previously [14]. Briefly, control and VLP(+Vpx) transduced or SAMHD1 KO cells were lysed in ice-cold 65% methanol and boiled at 95 °C for three minutes. The lysate was centrifuged, the pellet discarded, and the supernatant dried in a speed vacuum. The dried samples were rehydrated and used in the HIV-1 RT-based ^32^P-labeled primer extension assay to determine the cellular dNTP concentration. The extended and unextended primers were resolved by urea-PAGE, dried, and scanned using a Bio-Rad personal molecular imager. The data were quantified using Bio-Rad image lab 6.1, and the dNTP concentration was determined.

***Gene Expression Omnibus microarray***. Gene expression profiles in GSE4290 [31] and GSE16011 were downloaded from the NCBI Gene Expression Omnibus (GEO) database (http://www.ncbi.nml.nih.gov/geo/, accessed on 21 January 2022). The GSE4290 dataset contained 23 non-tumor brain tissues and 157 malignant glioma tumor tissues from patients pathologically diagnosed according to the WHO standard and was analyzed based on the GPL570 platform (Affymetrix GeneChip Human Genome U133 Plus 2.0 Array) [31]. A significant *p*-value was obtained using ANOVA pairwise comparison with Tukey’s post-hoc test. The GSE16011 dataset contained 8 non-tumor and 276 glioma samples, including 159 GBM samples. The data were analyzed using the GPL8542 platform (Affymetrix GeneChip Human Genome U133 Plus 2.0 Array) [32]. The TCGA dataset with samples containing LGG and GBM was downloaded and analyzed for IDH mutation, chromosome 1p/19q deletion, and SAMHD1 expression.

## 3. Results

### 3.1. SAMHD1 Is Highly Expressed in GBM

**Figure 1:**
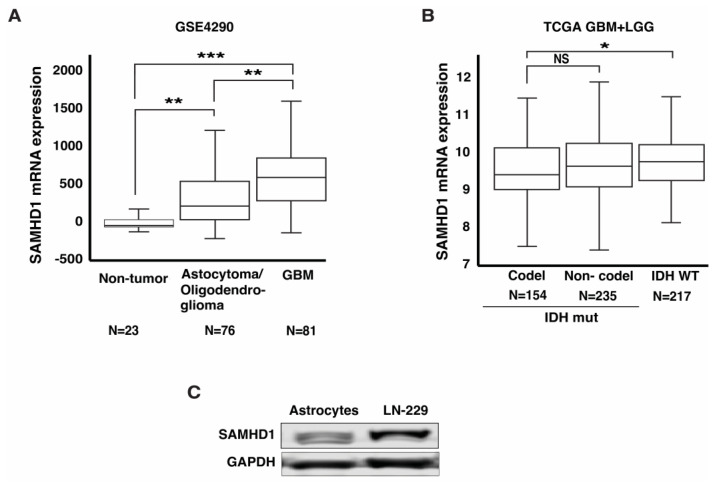
SAMHD1 is highly expressed in GBM. Data sets containing information from tumor and non-tumor samples were downloaded and analyzed for SAMHD1 expression. (**A**) GEO-GSE4290 data set [that contains 180 samples from 23 non-tumor, 76 astrocytoma and oligodendroglioma, and 81 GBM brain tissue were analyzed for SAMHD1 expression. (**B**) TCGA dataset with LGG and GBM samples with mutation information was divided into IDH mutation and 1p/19q codeletion (codel), IDH mutation and no 1p/19q codeletion (non-codel), and IDH WT. (**C**) SAMHD1 expression profile in LN-229 as compared to normal astrocytes. Statistical analysis (* ≤0.05, ** ≤0.01, and *** ≤0.001). Uncropped Western blot is presented in the Supplementary Materials.

DNA damage repair pathways have been extensively investigated to identify novel therapeutic targets for various cancers. γ-irradiation (IR) and temozolomide (TMZ) induce catastrophic cell death via DNA double-strand breaks (DSB). Thus, targeting the proteins involved in DSB repair could improve their anticancer efficacy. SAMHD1 promotes DSB repair through homologous recombination (HR), and several differentially expressed genes have been identified and implicated in the GBM response to DNA damage-inducing agents and patient prognosis [33]. Therefore, we evaluated SAMHD1 expression in malignant gliomas. To achieve this, the Gene Expression Omnibus (GEO) GSE4290 dataset, containing 23 non-tumor and 157 tumor samples from glioma patients [31], and GSE16011, which contains 276 glioma and 8 non-tumor brain tissue samples, were assessed [32]. As shown in Figure 1A and Supplementary Figure S1A, GBM samples express a significantly higher level of SAMHD1 compared to non-tumor brain samples in both datasets. The evaluation of malignant glioma genomic composition indicates that several differentially overexpressed genes in GBM promote tumor progression [34]. Therefore, we divided the GSE4290 glioma patient samples into WHO grade IV (GBM) and grades II and III (astrocytomas and oligodendrogliomas). The ANOVA yielded a significant *p*-value of 0.018, and in a pair-wise comparison with Tukey-Kramer’s post-hoc test, samples obtained from GBM patients showed a significantly elevated SAMHD1 level compared to both lower-grade gliomas and non-tumor brain samples (Figure 1A). The variation observed in gene expression was validated by Western blot analysis with lysates from normal astrocytes and two glioma cell lines, H4 (low-grade) and LN-229 (GBM) (Figure 1C and Supplementary Figure S1B). In the recent classification based on molecular parameters, glioma is categorized into subtypes with isocitrate dehydrogenase (IDH) mutation (IDH mut) and codeletion of chromosome 1 short arm (1p) and chromosome 19 long arm (19q) (IDH mut-codel) or IDH mut with no 1p/19q deletion (IDH mut non-codel). Current observations show that IDH wild type (IDH WT) gliomas have the poorest prognoses. Approximately 95% of primary GBM cases are IDH WT, whereas IDH WT low-grade glioma (LGG) is rare [5]. Thus, we analyzed TCGA data that contained both LGG and GBM samples with mutation profiles [35]. Interestingly, when dividing gliomas based on this classification, IDH WT gliomas expressed significantly higher SAMHD1 levels than IDH mut-codel, the least aggressive glioma (Figure 1B). Although a more extensive investigation could enhance the significance of our findings, the results from the patient tissue sample and cell line analyses suggest that GBM expresses a higher level of SAMHD1, indicating its importance in aggressive GBM pathogenesis.

### 3.2. Vpx-Mediated SAMHD1 Depletion Sensitizes GBM Cells to a PARP Inhibitor and TMZ

**Figure 2:**
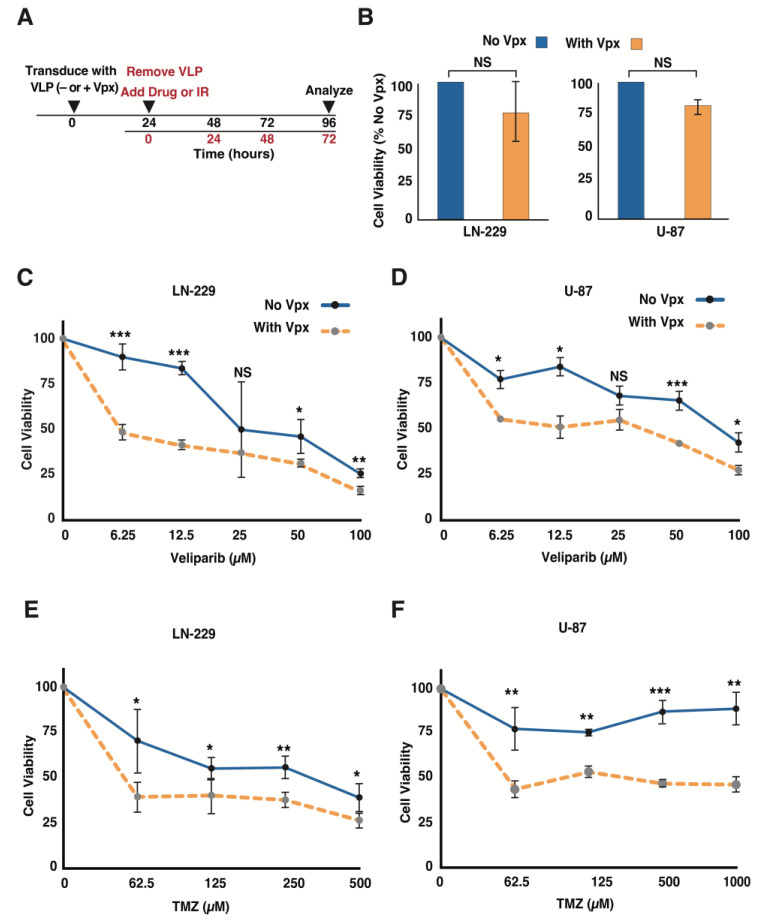
SAMHD1 depletion sensitizes glioma cells to DNA damage-inducing agents. (**A**) The diagram shows the workflow to determine the effect of Vpx-mediated SAMHD1 depletion on glioma cell sensitivity to DNA-damaging chemotherapeutic drugs. (**B**) LN-229 and U-87 cell lines were transduced with VLP(−Vpx) or (+Vpx), and viability was analyzed using AlamarBlue 96 h later. (**C**,**D**) GBM cell lines described in (**B**) were transduced with VLP(−VLP) or (+Vpx) and incubated at 37 °C for 24 h. Then, the cells were treated with Veliparib and grown for additional 72 h prior to cell viability analysis. The results are for LN-229 (**C**) and U-87 (**D**). (**E**,**F**) GBM cells were transduced with VLP(−Vpx) and (+Vpx) as described above, were incubated at 37 °C for 24 h, and treated with TMZ. A total of 72 h post-exposure to TMZ, cell viability was analyzed for LN-229 (**E**) and U-87 (**F**). The error bars represent a standard deviation of triplicates, and the asterisk is *p*-values (* ≤0.05, ** ≤0.01, and *** ≤0.001).

Elevated gene expression in tumors often indicates the importance of the gene and the pathway it promotes. The data presented above suggest that GBM expresses a higher level of SAMHD1, which localizes to the DNA damage site to promote DSB repair through HR [22,23]. However, SAMHD1’s role in the GBM response to DNA damage-inducing agents is unknown. The delivery of viral protein X (Vpx) into diverse cell types promotes proteasome-dependent SAMHD1 degradation, successfully depleting its intracellular levels [14]. Furthermore, we reported that Vpx and other depletion methods, such as siRNA, have similar counteracting effects on SAMHD1 function in HR [23]. Virus-like particles (VLPs) are formidable emerging therapeutic agents’ delivery systems suitable for in vitro and in vivo applications [36]. More importantly, VLPs are easy to utilize and have lower toxicity while achieving consistent and effective Vpx-mediated SAMHD1 depletion. Thus, we opted to use Vpx as a tool to deplete cellular SAMHD1. To accomplish this, we generated VLPs containing Vpx (VLP(+Vpx)), as previously described [30] and illustrated in Supplementary Figure S2A. The VLP titer was determined (Supplementary Figure S2B), and the minimal VLP(+Vpx) resulting in successful cellular SAMHD1 depletion was determined in the LN-229 and U-87 cell lines 24 h post-transduction (Supplementary Figure S2C,D). These cells were transduced with an equal amount of VLP(+Vpx) and VLP without Vpx (VLP(−Vpx)), media was changed 24 h later, and cell viability was determined 96 h post-transduction (Figure 2A). As shown in Figure 2B, both LN-229 and U-87 showed comparable growth at 96 h post-transduction with the minimum amount of VLP(+Vpx) required to induce notable SAMHD1 degradation (0.25 µg/mL) or equal amount of VLP(−Vpx). The deficiency of genes involved in DNA damage repair, particularly those supporting homologous recombination (HR), sensitizes various cancer cells, including GBM, to a poly (ADP-ribose) polymerase (PRAP) inhibitor (PARPi) [37,38]. Therefore, owing to its role in HR, we hypothesized that SAMHD1 depletion may sensitize GBM cells to PARPi. To test this, we depleted SAMHD1 using VLP(+Vpx) and assessed LN-229 and U-87 sensitivity to ABT888 (veliparib), a well-studied PARPi that has been investigated as a potential therapeutic agent for GBM [39]. As shown in Figure 2C,D, both LN-229 and U-87 cells showed significant sensitivity to veliparib following exposure to VLP(+Vpx). TMZ, a GBM chemotherapeutic agent that forms O(6)-methylguanine (O(6)MeG), adducts to cause cell death by inducing lesions believed to require mismatch repair (MMR). However, there is strong evidence that double-strand break repair pathways are also critical for resistance to O(6)MeG adducts and that inhibition of HR enhances cellular vulnerability to TMZ [40,41]. Thus, we investigated whether SAMHD1 depletion could sensitize malignant glioma cells to TMZ. Indeed, Vpx-mediated SAMHD1 depletion resulted in increased sensitivity of U-87 and LN-229 cells to TMZ (Figure 2E,F). These observations demonstrated that SAMHD1 plays an essential role in HR-mediated DNA damage repair and could promote resistance to TMZ in GBM. More importantly, it shows the potential synergistic effect of TMZ treatment and SAMHD1 depletion.

### 3.3. Delivery of Vpx into GBM Cells Causes Dose-Dependent Cell Growth Inhibition

**Figure 3:**
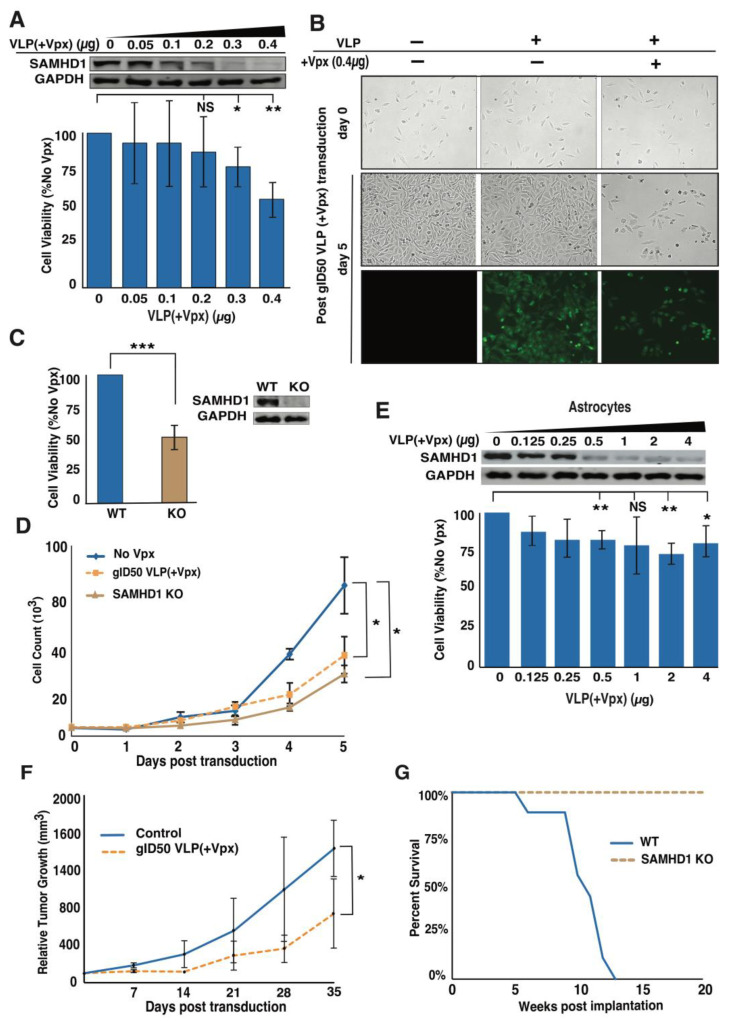
VLP(+Vpx) shows dose-dependent inhibition of malignant glioma cell growth. (**A**) LN-229 cells were transduced with varying concentrations of VLP(+Vpx). Then, 24 h post-transduction, cells were collected and analyzed for SAMHD1 degradation efficiency (top), while the rest were seeded on 96 well plates for AlamarBlue-based cell viability assay (bottom). (**B**) LN-229 cells were imaged before and five days post-transduction with gID50 VLPs with or without Vpx. (**C**) SAMHD1 knockout LN-229 (KO) was generated with an all-in-one CRISPR-Cas9 system, analyzed for cell growth, and compared to wild-type LN-229 (WT). (**D**) The growth kinetics of LN-229 cells transduced with gID50 VLP(+Vpx) and SAMHD1 KO cells were assessed by counting every day for five days. (**E**) Normal astrocytes were transduced with varying amounts of gID50 VLP(+Vpx), and growth was analyzed five days post-transduction. (**F**) LN-229 cells xenograft was subcutaneously established on both flanks of three Athymic Nude mice. After approximately one month of growth, tumor volume was measured and recorded as a baseline. The tumor on one side was injected with 0.1mL VLP(+Vpx) for three consecutive days and every week thereafter. The tumor volume was measured every week, and the result was plotted. (**G**) SAMHD1 KO (N = 8) or WT (N = 9) LN-229 cells were implanted into the brains of athymic mice, the survival was monitored, and the percent survival was plotted. The error bars represent a standard deviation of triplicates, and the asterisk is *p*-values (* ≤0.05, ** ≤0.01, and *** ≤0.001). Uncropped Western blots are presented in the Supplementary Materials.

Vpx-mediated SAMHD1 depletion is dose-dependent in differentiated and growth-arrested normal cells [42]. However, the dose-dependent Vpx-mediated SAMHD1 deficit and its effects have not yet been investigated in cancer cells, including GBM. Thus, we evaluated the impact of VLP(+Vpx) on LN-229 by escalating the minimum dose that was sufficient to cause considerable cellular SAMHD1 depletion (0.25 µg) (Supplementary Figure S2C). Vpx transduction suppressed cellular SAMHD1 levels, and subsequent GBM cell growth in a dose-dependent manner (Figure 3A). Interestingly, 0.4 µg/mL VLP(+Vpx) resulted in the highest SAMHD1 depletion 24 h post-transduction and caused about fifty percent reduced cell growth (Figure 3A,B). Thus, we used this dose as the 50% growth-inhibitory dose (gID50). An equal number of cells plated at the time of transduction (day 0) and the varying growth five days post-transduction (day 5) are shown in Figure 3B. Cells transduced with gID50 VLP(+Vpx) showed considerably lower cell growth. More importantly, cells exposed to similar doses of VLP(−Vpx) did not slow cell growth five days post-transduction (Figure 3B), demonstrating that the observed delayed cell growth was due to SAMHD1 depletion. Moreover, LN-229 cells transduced with VLP(−Vpx) exhibited a growth rate similar to mock control cells. To unequivocally show that the observed impaired cell growth was due to Vpx-mediated SAMHD1 depletion, we generated a SAMHD1 knockout (KO) LN-229 cell line using the CRISPR/Cas9 system. Equal numbers of wild-type (WT) and KO cells were seeded, and viability was assessed after five days. Similar to gID50 Vpx-mediated SAMHD1 depletion, SAMHD1 KO cells showed a 50% reduction in cell growth (Figure 3C). To further demonstrate impaired growth following SAMHD1 KO and Vpx-mediated depletion, we compared the cellular proliferation rate for five days. Both LN-229 and U-87 cells treated with gID50 VLP(+Vpx) and SAMHD1 KO cells grew significantly slower (Figure 3D and Supplementary Figure S3A). Although understanding the consequences of Vpx-mediated SAMHD1 degradation in cancer cells is essential for developing a potential therapeutic strategy, it is also critical to verify the effects of SAMHD1 loss on normal brain tissues. Thus, we evaluated the effect of Vpx-mediated SAMHD1 depletion on the growth of normal astrocytes that displayed lower SAMHD1 expression (Figure 1C). Interestingly, normal astrocytes displayed a high tolerance to VLP(+Vpx) exposure and did not achieve gID50 VLP(+Vpx) even at 4 µg/mL, which was 10-fold higher than the amount that achieved gID50 in LN-229 cells (Figure 3E). Furthermore, the lower-grade glioma cell line (H4) that displayed reduced SAMHD1 expression compared to LN-229 tolerated VLP(+Vpx) exposure, achieving gID50 at 5µg/mL (Supplementary Figures S1B and S3C).

To assess the effect of Vpx-mediated SAMHD1 depletion on GBM tumor progression, we established LN-229 cell xenografts on the left and right flanks of athymic nude mice. The tumor on one side was injected with VLP(+Vpx), and the other side received media, and both tumor volumes were monitored for 35 days. As shown in Figure 3F, the tumor that received VLP(+Vpx) grew slower than that of the control, supporting our tissue culture observations. Furthermore, we assessed the effect of SAMHD1 deficiency on mouse survival by implanting SAMHD1 WT LN-229 or SAMHD1 KO LN-229 cells into the brains of athymic nude mice and monitoring them over time. We found that mice implanted with SAMHD1-KO LN-229 survived longer than those implanted with SAMHD1 WT (Figure 3G). Previously, human GBM cell culture (HGCC) resources demonstrated that cultured patient-derived GBM cells (PDGC) have diverse proliferation rates. In addition, HGCC profiled the expression of genes associated with survival in a patient-derived mouse xenograft (PDX) model [43]. Our data suggested a high SAMHD1 expression in GBM and that depletion reduced cell growth. Therefore, we assessed whether low SAMHD1 expression is associated with reduced PDGC proliferation. As predicted, cultured GBM cells expressing lower SAMHD1 displayed a reduced proliferation rate. Similarly, in a mouse xenograft model, PDGC with a lower proliferation rate resulted in favorable survival (Supplementary Figure S3C,D). One of the well-established functions of SAMHD1 is its dNTPase activity [14]. Considering this, SAMHD1 depletion could increase cellular dNTP levels and possibly affect cell growth. Thus, we determined whether dNTP levels in GBM cells would be affected by SAMHD1 depletion. As shown in Supplementary Figure S3E, gID50 VLP(+Vpx)-mediated SAMHD1 depletion and CRISPR-mediated knockout resulted in an approximately 2-fold increase in the dNTP pool, comparable to non-GBM cancer cell line [23]. However, we did not observe notable changes in the cell cycle profile (Supplementary Figure S3F). The reduced cell proliferation could be due to slowed growth or induced apoptosis. Interestingly, upon assessing for the apoptosis by probing for cleaved caspase 3, we found a minimal cleavage 24 h post-transduction with VLP(+Vpx), which was considerable after 72 h (Supplementary Figure S3H). Although the specific mechanism involved in the gID50 VLP(+Vpx)-mediated reduced cell growth is out of the scope of this study, it could be due to induced apoptosis. These findings demonstrate that VLP(+Vpx)-mediated SAMHD1 depletion is dose-dependent, and the extent of SAMHD1 depletion correspondingly curbs GBM cell proliferation.

### 3.4. gID50 VLP(+Vpx)-Induced SAMHD1 Depletion Impairs Cellular DNA Damage Repair Potential

**Figure 4:**
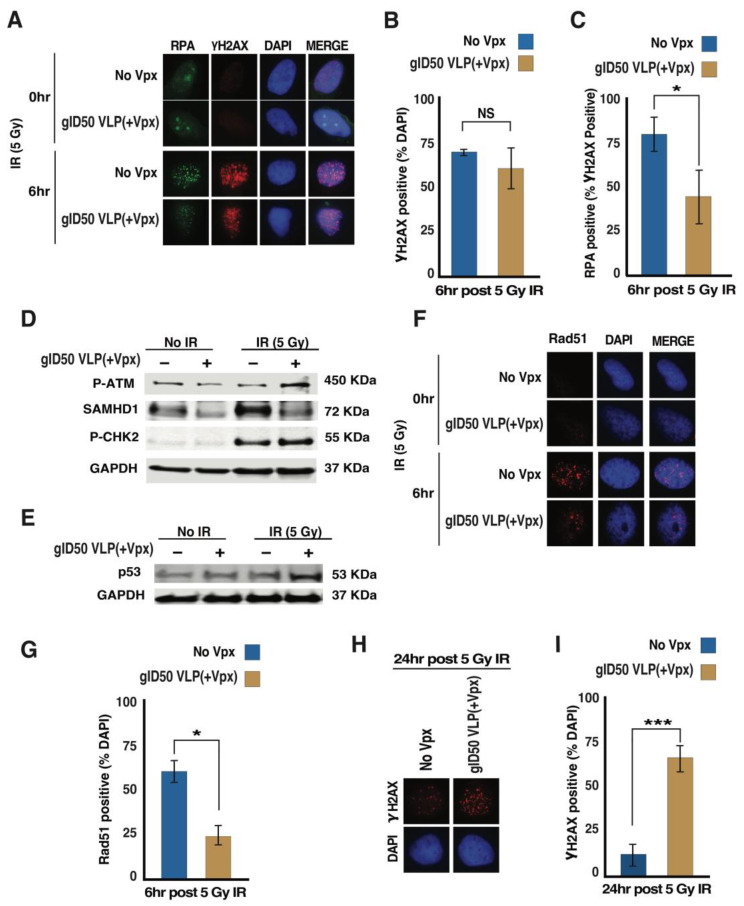
SAMHD1 depletion delays DNA damage repair in GBM cells (**A**) LN-229 cells exposed to gID50 VLP(+Vpx) or VLP(−Vpx) for 24 h were irradiated with the indicated dose. Four hours post-irradiation, cells were fixed, probed for RPA70, γH2AX, and DAPI prior to staining and analyzed for foci. (**B**,**C**) A set of fifty DAPI positive cells were randomly counted three times and analyzed for γH2AX and RPA70 foci. The quantified data is presented as (**B**) percent γH2AX positive of the DAPI, and (**C**) the percent RPA70 not impaired of γH2AX positive cells. (**D**,**E**) Evaluation of P-ATM and PCHK2 6 h post-exposure to 5 Gy irradiation. (**F**,**G**) An immunofluorescence assay showing the evaluation of Rad51 accumulation at the damage sire following exposure to 5 Gy IR. (**H**,**I**) The evaluation of remaining γH2AX 24 h post exposure to 5 Gy irradiation in LN-229 transduced with gID50 VLP(+Vpx) or VLP(−Vpx). Presented are (**H**) representative images and (**I**) quantified results. The error bars represent a standard deviation of triplicates, and the asterisk is *p*-values (* ≤0.05, and *** ≤0.001). Uncropped Western blots are presented in the Supplementary Materials.

The GBM total gene expression analysis indicates that proteins encoded by various overexpressed genes support tumor progression and therapy resistance by promoting diverse pathways, including DNA damage repair [34,44]. SAMHD1 depletion enhanced the sensitivity of GBM cells to PARPi and TMZ, which are both DNA damage-inducing agents (Figure 2). The increased sensitivity of GBM cells to PARPi, following SAMHD1 depletion, indicated SAMHD1 role in HR-mediated DNA double-strand break (DSB) repair in GBM (Figure 2A,B). Thus, we examined whether the initial step of HR-mediated DSB repair is affected in gID50 VLP(+Vpx)-transduced LN-229 cells by monitoring the accumulation of replication protein A (RPA), a single-stranded DNA-binding protein, and a well-accepted DNA-end resection marker that emerges at the initial stage of HR. As shown in Figure 4A, RPA localization to DSB sites was impaired in gID50 VLP(+Vpx) transduced GBM cells following exposure to 5 Gy IR. Upon quantification, we found that at 6 h post-irradiation, cells transduced with gID50 VLP(−Vpx) or (+Vpx) displayed comparable γH2AX foci, confirming equal IR-induced DSBs (Figure 4B). However, in LN-229 transduced with gID50 VLP(+Vpx), a significantly higher percentage of cells with γH2AX foci displayed impaired RPA localization to damage sites (Figure 4C). This provides mechanistic evidence for how SAMHD1 depletion potentiated LN-229 and U-87 sensitivity to PARPi and TMZ (Figure 2C–F). Six hours post IR exposure, ATM and CHK2 phosphorylation remained higher in LN-229 cells transduced with gID50 VLP(+Vpx) (Figure 4D). In addition, the IR-induced p53 expression was higher 6 h post-exposure in LN-229 transduced with gID50 VLP(+Vpx) (Figure 4E). Autophosphorylation of ATM is induced at the early stage of DSBs and tapers within a few hours [45,46]. In our hand, phosphorylated ATM was induced at 30 min post IR exposure and diminished about two hours later (Supplementary Figure S4A). Thus, the elevated phosphorylated ATM and higher p53 expression 6 h post-IR exposure suggest the persistent presence of DSBs in SAMHD1 depleted cells. If the DNA end resection is impaired, theoretically, the downstream process of homology searching, which is mediated by Rad51 and associated proteins, will also be reduced. Indeed, our evaluation confirmed the diminished accumulation of Rad51 at the DNA damage site in cells transduced with gID50 VLP(+Vpx) and exposed to 5 Gy IR, demonstrating the potential overall impairment of HR (Figure 4F,G). γH2AX foci is a well-established DNA DSB marker, and the numbers of γH2AX foci correspond to the extent of induced and lingering DSBs. γH2AX foci diminish when DSB lesions are successfully repaired, thus providing a measurable tool to assess the repair kinetics [47]. Accordingly, to assess the extent of repair, we compared the residual γH2AX foci 24 h post-5 Gy IR exposure. Interestingly, LN-229 cells transduced with gID50 VLP(−Vpx) displayed significantly reduced γH2AX foci (Figure 4H,I), suggesting a successful DSB repair. However, cells transduced with gID50 VLP(+Vpx) have higher γH2AX foci that continue to linger, revealing the critical delay of DSB repair. This result is consistent with the impaired RPA localization to the DNA damage sites 6 h post-5Gy IR exposure (see above). These findings strongly suggest that SAMHD1 promotes DSB repair in GBM and provides a mechanistic explanation for how SAMHD1 depletion sensitizes GBM cells to DNA damage-inducing agents.

### 3.5. Exposing Malignant Glioma Cells to gID50 VLP(+Vpx) Enhances Their Sensitivity to TMZ and Ionizing Radiation (IR)

**Figure 5:**
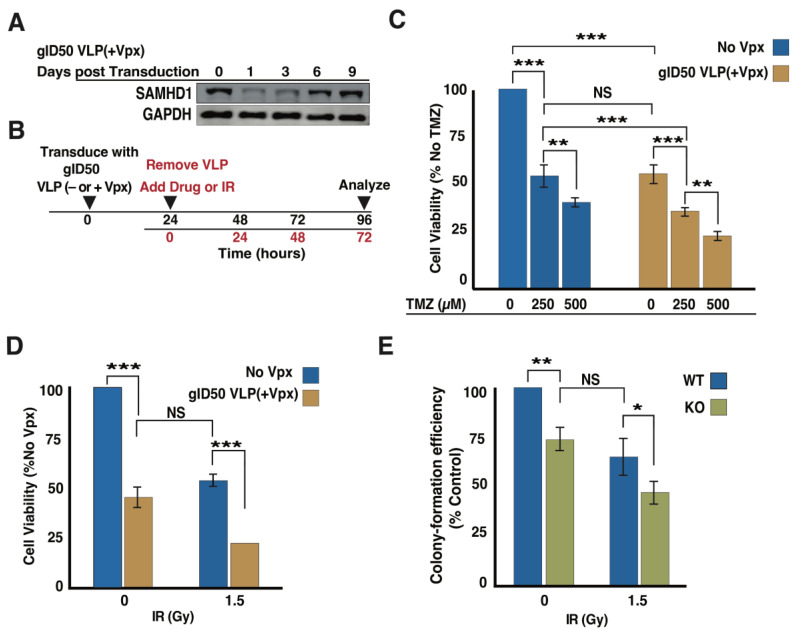
Exposure to ID50 VLP(+Vpx) further sensitizes GBM cells to DNA damage inducing agents. (**A**) LN-229 cells were exposed to gID50 VLP(+Vpx) for 24 h and assessed for SAMHD1 depletion up to 9 days post-transduced. (**B**) The schematic description of experimental outline for gID50 VLP(+Vpx) transduction and drug or IR treatment. (**C**) LN-229 cells transduced with gID50 VLP(+Vpx) for 24 h were treated with 250 and 500 µM TMZ and the sensitivity was analyzed four days post exposure to TMZ. (**C**,**D**) Cells were analyzed for sensitivity to indicated dose (Gray) of IR in gID50 VLP(+Vpx) transduced (**D**) or SAMHD1 KO (**E**) LN-229. The error bars represent a standard deviation of triplicates and asterisk are *p*-values (* ≤0.05, ** ≤0.01, and *** ≤0.001). Uncropped Western blot is presented in the Supplementary Materials.

Although transduction by gID50 VLP(+Vpx) resulted in reduced GBM cell growth and impaired DSB repair, it was not clear whether VLP(+Vpx)-mediated SAMHD1 reduction alters the response to DNA damage-inducing agents. We assumed that the combination of gID50 VLP(+Vpx) could improve the sensitivity to conventional GBM treatments, TMZ, and IR. To evaluate this possibility, we performed cell viability and clonogenic assays. We first assessed the duration of gID50 VLP(+Vpx) SAMHD1 depletion post-transduction. As shown in Figure 5A, SAMHD1 depletion was optimal at 24 h and lasted for up to 72 h post-transduction in LN-229 cells. Cellular SAMHD1 levels gradually recovered and reached an average level on day nine. Our data suggested that 24 h post-transduction was the optimal time to treat cells with DNA damage-inducing agents (Figure 5B). gID50 VLP(+Vpx)-exposed cells displayed a significantly reduced proliferation rate and were further sensitized to TMZ compared to cells treated with VLP(−Vpx) (Figure 5C). Interestingly, 250 µM TMZ treatment and transduction with gID50 VLP(+Vpx) showed a comparable effect on LN-229 cell viability. However, the combination of these two treatments resulted in significantly reduced cell viability compared to when they were administered separately. Similarly, the combination of gID50 VLP(+Vpx) and IR resulted in enhanced sensitivity (Figure 5D). In agreement with the results obtained from gID50 VLP(+Vpx), SAMHD1-KO LN-229 cells showed markedly enhanced sensitivity when combined with IR (Figure 5E). These results indicated that gID50 VLP(+Vpx)-mediated SAMHD1 depletion, SAMHD1 KO, TMZ, and IR affected the same pathway confirming the synergistic effect demonstrated in Figure 2. Our study demonstrated that in GBM, SAMHD1 depletion curbs cell growth, impairs DSB repair, and potentiates sensitivity to DNA-damaging agents. Furthermore, this suggests that targeting SAMHD1 could be a feasible anti-GBM strategy that improves TMZ and RT efficacy.

## 4. Discussion

**Figure 6:**
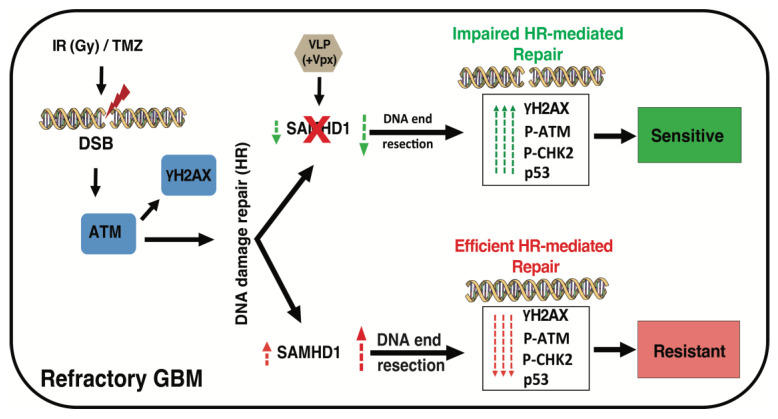
The schematic diagram showing GBM cell response to IR and TMZ. In the GBM cells, IR or TMZ induces DSB, activating DNA damage response genes such as ATM, CHK2, and p53. Consequently, genes involved in HR will facilitate effective repair. In the GBM cells expressing a high level of SAMHD1, there will be efficient HR-mediated DNA damage repair to counteract the effect of TMZ or IR, leading to resistance. However, when transduced with VLP(+Vpx), SAMHD1 depletion will impair HR, marked by lingering DNA damage response genes activation. The impaired DAN damage repair improves GBM cell sensitivity to TMZ or IR.

Conventional treatment for GBM includes radiation therapy (RT) combined with chemotherapeutic alkylating agents, such as TMZ. These treatments cause GBM cell death, partly through the induction of DNA double-strand break (DSB). Thus, DSB repair pathways have been extensively studied to develop novel treatments or improve the efficacy of currently available treatment options. Several therapeutic targets have been proposed, and some are currently in clinical trials (reviewed in [44]). Nevertheless, the median survival time for GBM remains at approximately 15 months, and there has been no breakthrough in overcoming this clinical barrier. Therefore, there is an urgent need to identify novel therapeutic targets and more efficient treatment strategies. Several differentially expressed genes have been identified and implicated in the GBM response to DNA damage-inducing agents [48]. We found that GBM tumors and GBM-derived cancer cell lines express a high level of SAMHD1, demonstrating its potential importance for this highly lethal cancer. Previous findings implicated SAMHD1 in diverse cellular processes, including DSB repair and R-loop resolving [20,21,23]. Therefore, a high level of SAMHD1 could contribute to resistance to DSB-inducing agents, and its depletion could sensitize GBM cells (Figure 6). Indeed, SAMHD1 depletion potentiated GBM cell sensitivity to Veliparib, a well-established PARP1 inhibitor. PARP1 is involved in DNA single-strand break repair and PARP1 inhibition results in the accumulation of DSBs after the collapse of the replication forks. Thus, an inhibited PARP1 function combined with an impaired DSB repair pathway leads to the accumulation of DSBs, resulting in enhanced therapeutic sensitivity. We showed that SAMHD1 depletion sensitizes GBM cells to TMZ, a currently available GBM treatment. The enhanced sensitivity to Veliparib and TMZ following SAMHD1 depletion confirms SAMHD1’s critical role in DSB repair in GBM, most likely by promoting homologous recombination (HR). This notion is supported by the impaired DSB repair following SAMHD1 depletion, as presented in Figure 4.

Impaired GBM cell growth following Vpx-mediated SAMHD1 depletion further highlights the relevance of its elevated expression in GBM. Interestingly, less aggressive brain tumors, such as astrocytoma and oligodendroglioma, showed lower SAMHD1 expression than GBM, the most aggressive malignant glioma characterized by a much higher proliferation rate (Figure 1A). This observation is supported by patient-derived GBM cell lines, in which higher proliferation correlated with elevated SAMHD1 expression (Supplementary Figure S3), consistent with a recent report that demonstrated U2OS cell slow proliferation following SAMHD1 knockdown [21]. However, another study has demonstrated that SAMHD1 knock-out ThP1 cell, a human monocytic cell line derived from an acute monocytic leukemia patient, exhibited an increased proliferation [49]. These findings and the work presented in this study demonstrate that the cellular response to SAMHD1 deficiency could differ depending on the cancer type. Despite impaired cell growth, we did not observe a significant change in the cell cycle profile following SAMHD1 depletion or SAMHD1 KO (Supplementary Figure S3F). The reduced cell proliferation without inducing a notable change in the cell cycle profile could result from a delay in at least one of the cell cycle phases, as previously demonstrated [50]. In the SAMHD1-depleted GBM cells, the lack of notable change in the cell cycle profile strongly suggests a delay rather than arrest. A more robust cell cycle analysis supported by molecular analysis of cell cycle-specific proteins could shed light on this matter. In addition, it is conceivable that the slight change in cell cycle change could be missed due to the sensitivity of the assay utilized. Since we utilized a viral vector to deliver Vpx into cells, it is critical to determine whether viral particles, including Vpx, elicit impaired cell growth through mechanisms other than SAMHD1 depletion. Treatment with VLP(−Vpx) partly addresses this concern, and observations in SAMHD1 KO cells confirm that SAMHD1 depletion is a crucial contributor to impaired growth. Nevertheless, systematic investigation to evaluate the potential contribution of Vpx (in addition to SAMHD1 depletion) and other viral proteins contained in VLP using meticulous cell cycle analysis would provide a more complete picture. There is a rationale to pursue and further clarify the SAMHD1 expression and function because our observation in GBM tumor samples, patient-derived GBM cell lines, and established GBM cell lines strongly indicate that SAMHD1 expression status renders GBM more malignant and refractory. We found that Vpx-mediated SAMHD1 depletion was dose-dependent and inhibited GBM cell growth in a dose-dependent manner (Figure 3A). The restored endogenous SAMHD1 expression following VLP(+Vpx) treatment suggests that Vpx could be cleared or modified to suppress its function. Furthermore, even at the highest concentration examined, Vpx could not deplete SAMHD1 completely. This could be due to different amounts of Vpx cells received. In this case, the population that received lower Vpx may contribute to the observed remaining SAMHD1. On the other hand, in some populations, SAMHD1 could be resistant to Vpx-mediated degradation due to sequestration or post-translational modification. Thus, more work is needed to fully understand Vpx regulation and its stability in cancer cells. Furthermore, careful consideration of timing is needed when utilizing this system for SAMHD1 depletion.

Treatment with gID50 VLP(+Vpx) alone delayed the growth of GBM cells, exhibiting an effect similar to that of 250 µM TMZ or 2 Gy IR. This finding, combined with the results from the LN-229 mouse xenograft model, which showed decreased tumor growth following VLP(+Vpx) treatment (Figure 3), demonstrated the potential benefit of targeting SAMHD1 in GBM. The combination of 250 µM TMZ with gID50 VLP(+Vpx) resulted in significantly higher toxicity than when administered alone. This result demonstrates the synergistic benefit of combining VLP(+Vpx) with conventional DNA-damaging GBM treatments. The gID50 VLP(+Vpx) treatment showed slightly better sensitivity than SAMHD1 KO (Figure 5D,E). Although a more controlled study is necessary, this indicates the possibility that GBM cells adapt to SAMHD1 loss by activating a potential alternative mechanism for known SAMHD1 functions. Thus, a sudden SAMHD1 loss, which better represents transient SAMHD1 depletion, could potentiate a higher sensitivity. In contrast, the prolonged loss of SAMHD1, which could result from natural SAMHD1 deficiency or mutation, may have a moderate effect.

The potential in vitro and in vivo application of VLPs and the high specificity of Vpx to SAMHD1 make the combination a more attractive strategic option. Therapeutic approaches involving targeted, induced protein degradation are still emerging, and much remains to be uncovered. However, its potential as an effective tool is beginning to be appreciated. To this end, some of the approaches investigated and proposed for cellular protein depletion include inhibiting deubiquitinases to elevate proteasomal degradation (reviewed in [51]) and utilizing small molecules to hijack the ubiquitin ligase complex (reviewed in [52]). However, these methods are broad and lack protein specificity. However, Vpx-mediated SAMHD1 depletion could provide better safety and specificity, and these technical advantages could make Vpx-mediated SAMHD1 degradation a formidable candidate for translational application. Our present study in cell lines and animal models shows that VLP(+Vpx) can be used as a therapeutic tool in GBM in vivo (Figure 3F,G). However, optimization of the delivery tool to allow for penetration of the blood-brain barrier and to enhance anti-GBM specificity might be needed prior to advancing VLP(+Vpx) for clinical application. A recent study has shown that Vpx induces an innate immune response independent of SAMHD1 degradation [53], indicating a need for a careful evaluation of VLPs and Vpx activities prior to considering their use in clinical setting. On the promising side the tolerance of normal astrocytes to VLP(+Vpx) treatment shows a potentially lower effect on normal cells or tissue around the GBM.

## 5. Conclusions

The presented study provides compelling evidence for considering SAMHD1 as a plausible novel therapeutic target and VLP(+Vpx) as a therapeutic tool, particularly to enhance the efficacy of TMZ and RT in GBM. However, a broader study could be helpful in unequivocally demonstrating the feasibility of targeting SAMHD1 to benefit GBM patients.

## Data Availability

The data that support the findings of this study are available from the corresponding author upon reasonable request.

## Supplementary Materials

The following supporting information can be downloaded at: https://www.mdpi.com/article/10.3390/cancers14184490/s1. Figure S1: SAMHD1 is highly expressed in GBM. (A) GSE16011 dataset that contains 159 GBM and 8 non-tumor brain tissue were analyzed for SAMHD1 expression. (B) Three established cell line H4, representing low grade glioma, and LN-229 and U-87, representing GBM, were analyzed for SAMHD1 level. Asterisks represent the *p*-values (* ≤ 0.05, ** ≤ 0.01, and *** ≤ 0.001); Figure S2: SAMHD1 is successfully deleted by delivering Vpx into GBM cells (A) Schematic diagram describing generation of Viral-like particles (VLPs) from 293 cells after co-transfection with VSVG and packaging plasmids that does not encode for Vpx(−Vpx) or encodes for Vpx(+Vpx). (B) The titration of VLPs to determine a quantity of particles to be used. The graph is a representation of the absorbance corresponding to the concentration of known standard. The measured absorbance value for the generated VLP(−Vpx) and (+Vpx) are presented in the graph. (C–D) SAMHD1 degradation efficiency of varying quantity of VLP(+Vpx) in LN-229 (C) and U-87 (D) cell lines; Figure S3: SAMHD1 expression level correlated with cell proliferation. (A) Five days-long U-87 growth was assessed post 24-hour gID50 VLP(+Vpx) exposure. (B) H4 cells were transduced with varying concentrations of VLP(+Vpx). Then, 24 h post-transduction, cells were collected and analyzed for SAMHD1 degradation efficiency (top), while the rest were seeded on 96 well plate for AlamarBlue-based cell viability assay (bottom). (C–D) The dataset was obtained from the HGCC database and analyzed. (C) The SAMHD1 expression level in patient-derived glioblastoma cells (PDGC) with low and not low proliferation quartile was compared by a one-tailed student T-test. (D) Survival profile of PDGC mice xenograft. The implanted PDGC cells were divided into low and not low proliferation rates and analyzed. (E) Cellular dNTP was extracted from gID50 VLP(+Vpx) and KO LN-229. The extract was assessed with HIV-1 RT-based primer extension assay. The product was resolved (top), and concentration was determined after quantification (bottom). (F) The cell cycle profile of gID50 VLP(+VPx) transduced and KO LN-229 (G) gID50 VLP(+Vpx) transduced LN-229 cells were evaluated for Caspase 3 cleavage 24 and 72 h post-transduction. *p*-values (* ≤ 0.05, ** ≤ 0.01, and *** ≤ 0.001); Figure S4: ATM is phosphorylation. LN-229 cells were exposed to 5 Gy IR and lysed at different times between 30 minutes and 6 hours. The lysate was resolved and probed for indicated genes and ATP phosphorylation; Table S1: Primary Antibodies; Table S2: Secondary Antibodies.
