# Supplementary material for: Viral Particle-Mediated SAMHD1 Depletion Sensitizes Refractory Glioblastoma to DNA-Damaging Therapeutics by Impairing Homologous Recombination
Source: Cancers (Basel). 2022 Sep 16;14(18):4490. doi: 10.3390/cancers14184490 (PMC9497202; doi:10.3390/cancers14184490)

cancers

MDPI

Supplementary Files,

Viral particle-mediated SAMHD1 depletion sensitizes refractory glioblastoma to DNA-damaging therapeutics by impairing homologous recombination.

Waaqo Daddacha[1,2] *, Dominique Monroe[1,2], Kristen Carver[1], Edidiong Usoro[1,2], Ahmet Alptekin[2], Hongyan Xu[3], Satoru Osuka[4], Ali S Arbab[1,2], and Daitoku Sakamuro[1,2]

1 Department of Biochemistry and Molecular Biology, Medical College of Georgia, Augusta University, Augusta, GA 30912
2 Georgia cancer center, Augusta University, Augusta GA 30912.
3 Department of Population Health Sciences, Medical College of Georgia, Augusta University, GA 30912.
4 Department of Neurosurgery, Heersink School of Medicine, The University of Alabama, Birmingham, AL, 35233.
* Correspond t Dr. Waaqo Daddacha at : wdaddacha@augusta.edu; Tel. 706721 0272

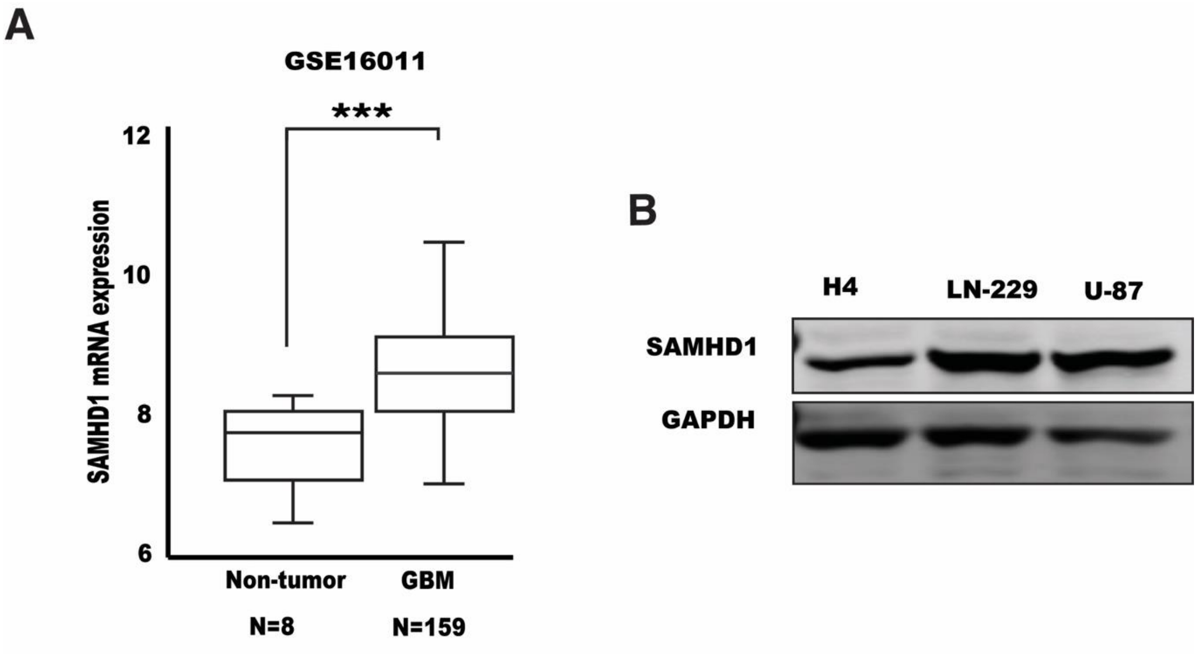

Supplementary Figure S1. SAMHD1 is highly expressed in GBM. (A) GSE16011 dataset that contains 159 GBM and 8 non-tumor brain tissue were analyzed for SAMHD1 expression. (**B**) Three established cell line H4, representing low grade glioma, and LN-229 and U-87, representing GBM, were analyzed for SAMHD1 level. Asterisks represent the p-values (*<=0.05, **<=0.01, and ***<=0.001).

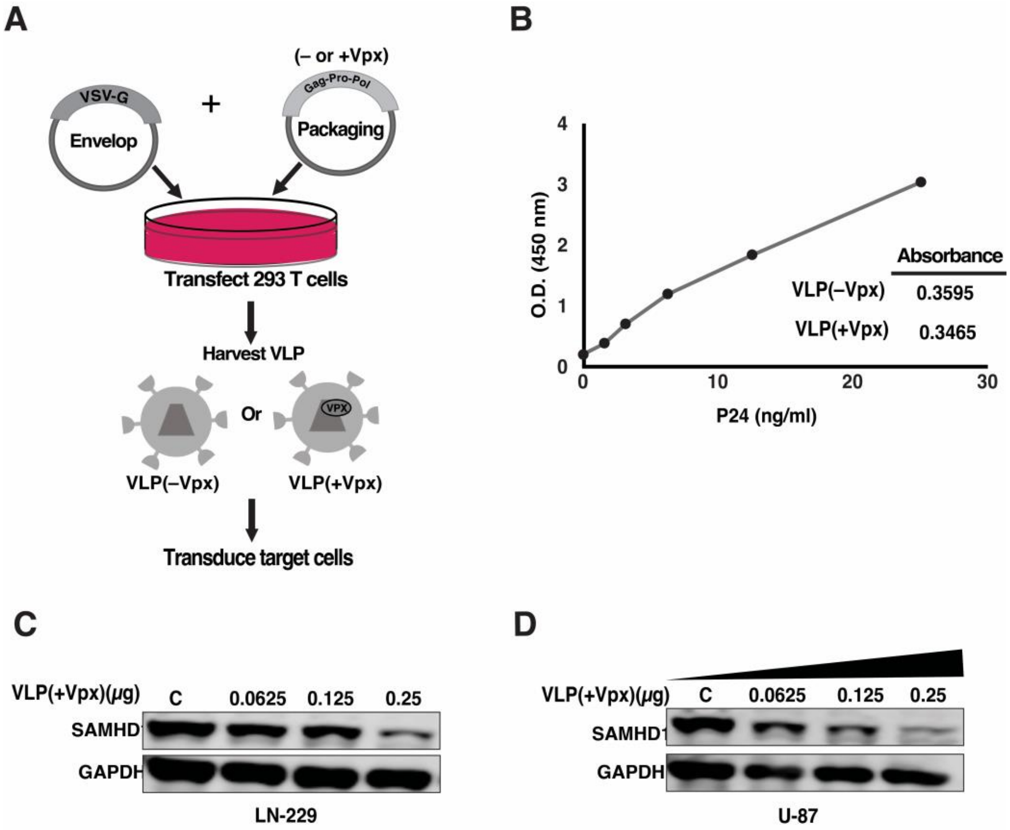

Supplementary Figure S2, SAMHD1 is successfully deleted by delivering Vpx into GBM cells (**A**) Schematic diagram describing the generation of Viral-like particles (VLPs) from 293 cells after co-transfection with VSVG and packaging plasmids that do not encode for Vpx (-Vpx) or encodes for Vpx (+Vpx). (**B**) The titration of VLPs to determine the quantity of particles to be used. The graph is a representation of the absorbance corresponding to the concentration of a known standard. The measured absorbance value for the generated VLP (-Vpx) and (+Vpx) are presented in the graph. (**C-D**) SAMHD1 degradation efficiency of varying quantity of VLP(+Vpx) in LN-229 (**C**) and U-87 (**D**) cell lines.

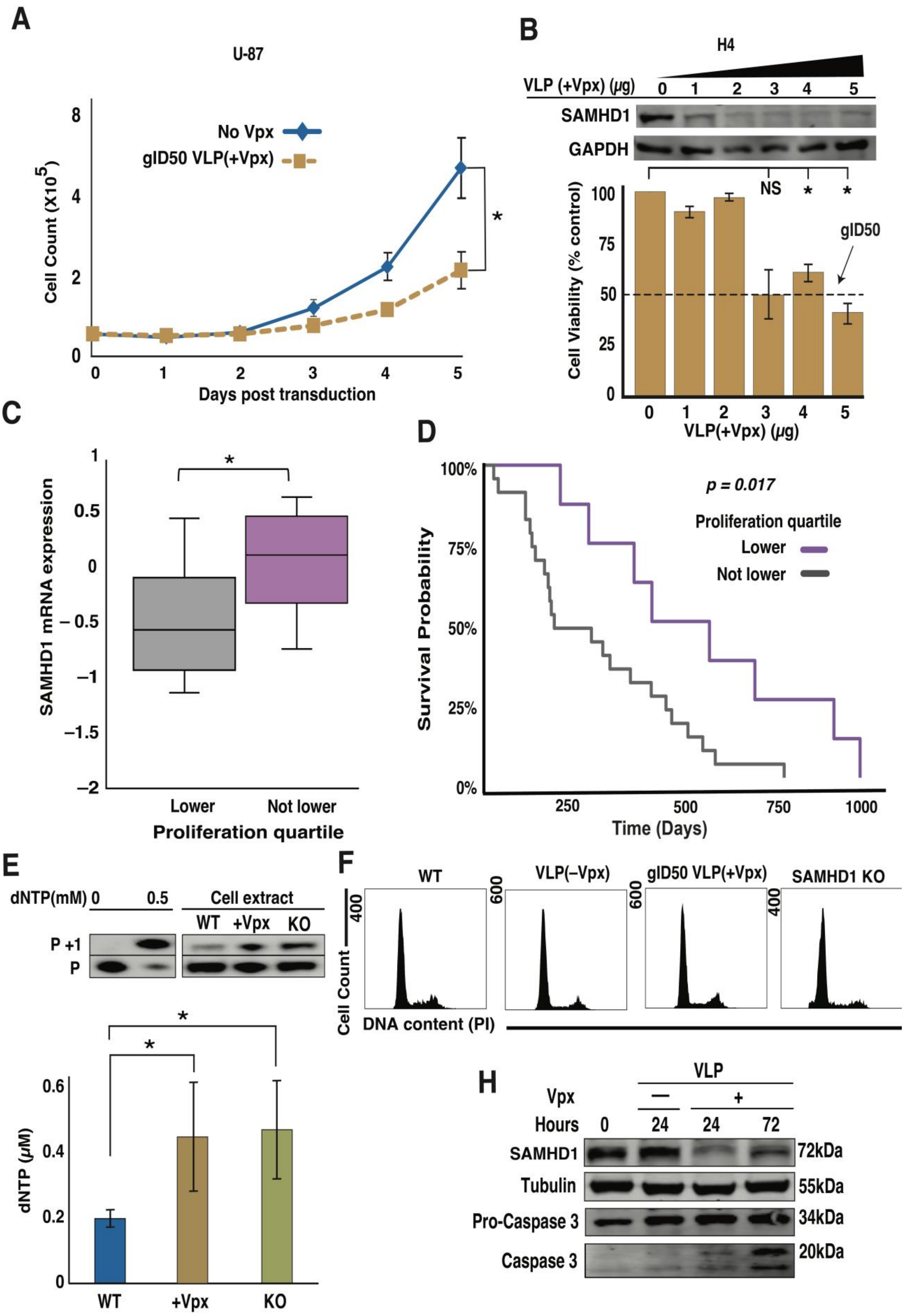
A
U-87
No Vpx
gID50 VLP(+Vpx)
Cell Count (X10^5)
Days post transduction
B
H4
VLP (+Vpx) (μg)
SAMHD1
GAPDH
NS
gID50
Cell Viability (% control)
VLP(+Vpx) (μg)
C
SAMHD1 mRNA expression
Lower
Not lower
Proliferation quartile
D
p = 0.017
Proliferation quartile
Lower
Not lower
Survival Probability
Time (Days)
E
dNTP(mM) 0 0.5
Cell extract
WT +Vpx KO
P +1
P
dNTP (μM)
WT
+Vpx
KO
F
WT
VLP(−Vpx)
gID50 VLP(+Vpx)
SAMHD1 KO
Cell Count
DNA content (PI)
H
VLP
Vpx
Hours
SAMHD1
Tubulin
Pro-Caspase 3
Caspase 3
72kDa
55kDa
34kDa
20kDa

Supplementary Figure S3, SAMHD1 expression level correlated with cell proliferation. (**A**) Five days-long U-87 growth was assessed post 24 h gID50 VLP(+Vpx) exposure. (**B**) H4 cells were transduced with varying concentrations of VLP(+Vpx). Then, 24 hours post-transduction, cells were collected and analyzed for SAMHD1 degradation efficiency (top), while the rest were seeded on 96 well plate for AlamarBlue-based cell viability assay (bottom). (**C-D**) The dataset was obtained from the HGCC database and analyzed. (**C**) The SAMHD1 expression level in patient-derived glioblastoma cells (PDGC) with low and not low proliferation quartile was compared by a one-tailed student T-test. (**D**) Survival profile of PDGC mice xenograft. The implanted PDGC cells were divided into low and not low proliferation rates and analyzed. (**E**) Cellular dNTP was extracted from gID50 VLP(+Vpx) and KO LN-229. The extract was assessed with HIV-1 RT-based primer extension assay. The product was resolved (top), and concentration was determined after quantification (bottom). (**F**) The cell cycle profile of gID50 VLP(+VPx) transduced and KO LN-229. p-values (*<=0.05, **<=0.01, and ***<=0.001).

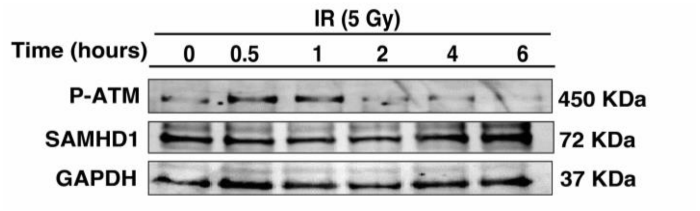

Supplementary Figure S4, ATM is phosphorylation. LN-229 cells were exposed to 5 Gy IR and lysed at different times between 30 minutes and 6 hours. The lysate was resolved and probed for indicated genes and ATP phosphorylation.

Table S1: Primary Antibodies

Name	Species	Company	Catalog #
SAMHD1	Rabbit	Proteintech	12586-1-ap
SAMHD1	Mouse	Origene	TA502024
P-ATM	Mouse	Cell Signaling	4526S
GAPDH	Mouse	Santa Cruz	sc-47724
GAPDH	Rabbit	Cell Signaling	2118S
P-Chk2	Rabbit	Cell Signaling	2197S
p53	Rabbit	Santa Cruz	sc-6243
RPA70	Rabbit	Cell Signaling	2267S
yH2AX	Mouse	Millipore	05-636
pRPA32 (S4/S8)			

Table S2: Secondary Antibodies

Name	Species	Company	Catalog #
Anti-mouse IgG Alexa 555	Donkey	Invitrogen	A31570
Anti-rabbit IgG-Alexa 555	Donkey	Invitrogen	A31572
Anti-mouse IgG- Alexa 488	Donkey	Invitrogen	A21202
Anti-rabbit IgG-Alexa 488	Donkey	Invitrogen	A21206

Raw Western blots

Figure 1C

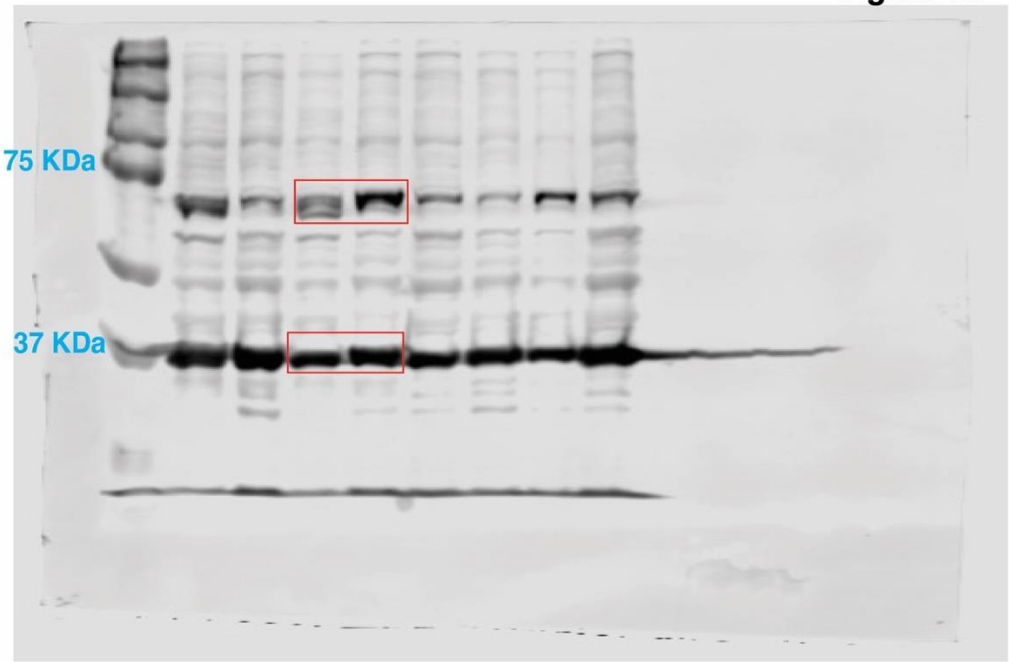

Figure 3A

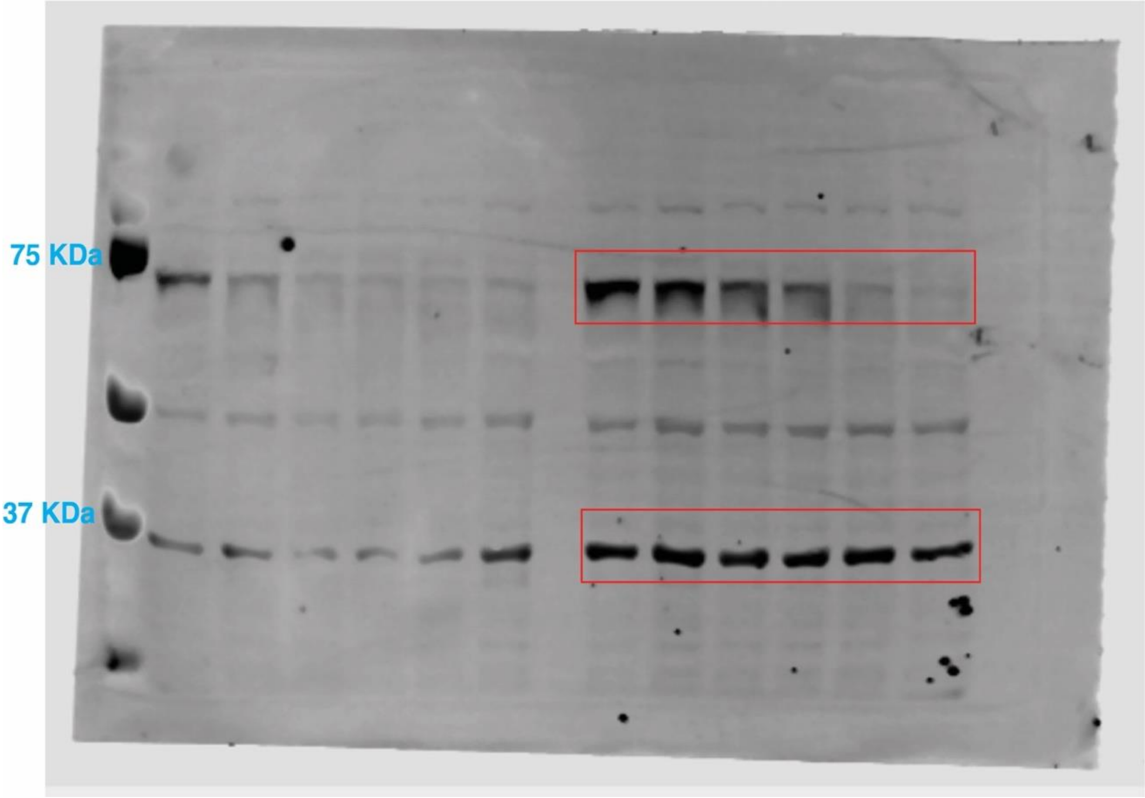

Figure 3C

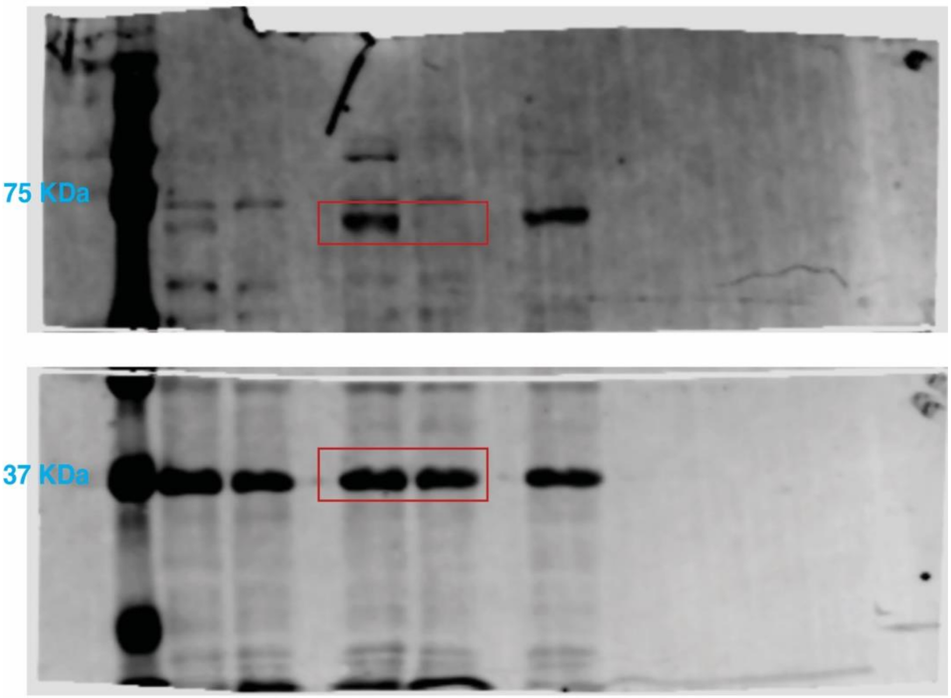

Figure 3E

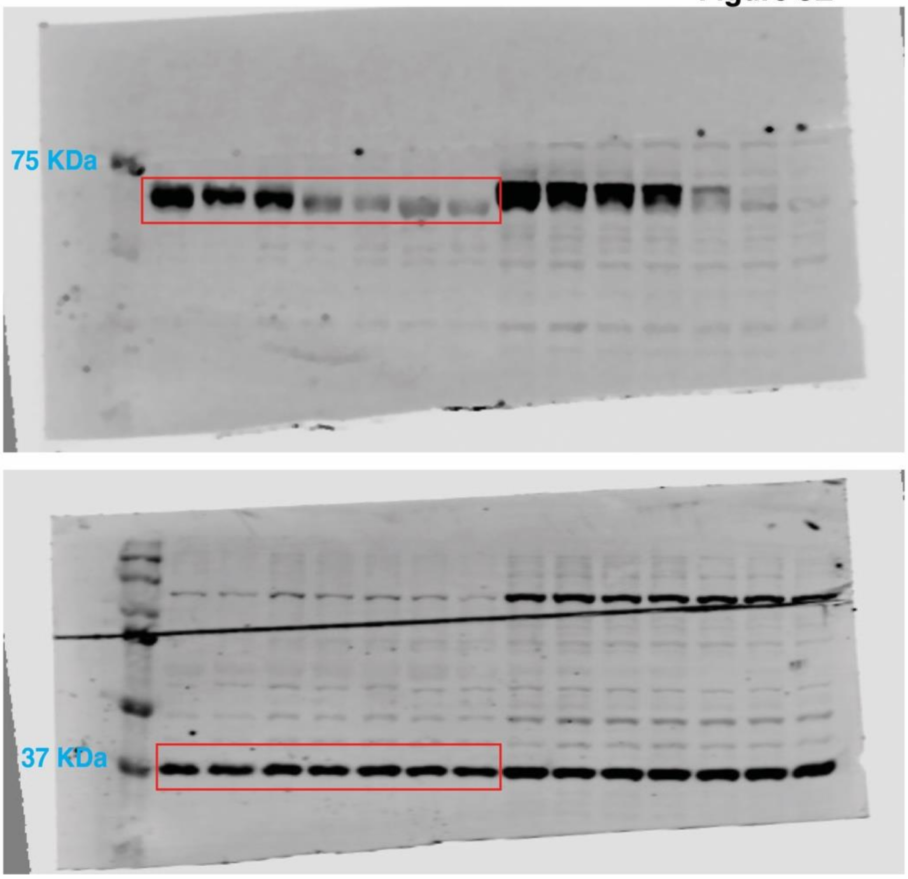

Figure 4D

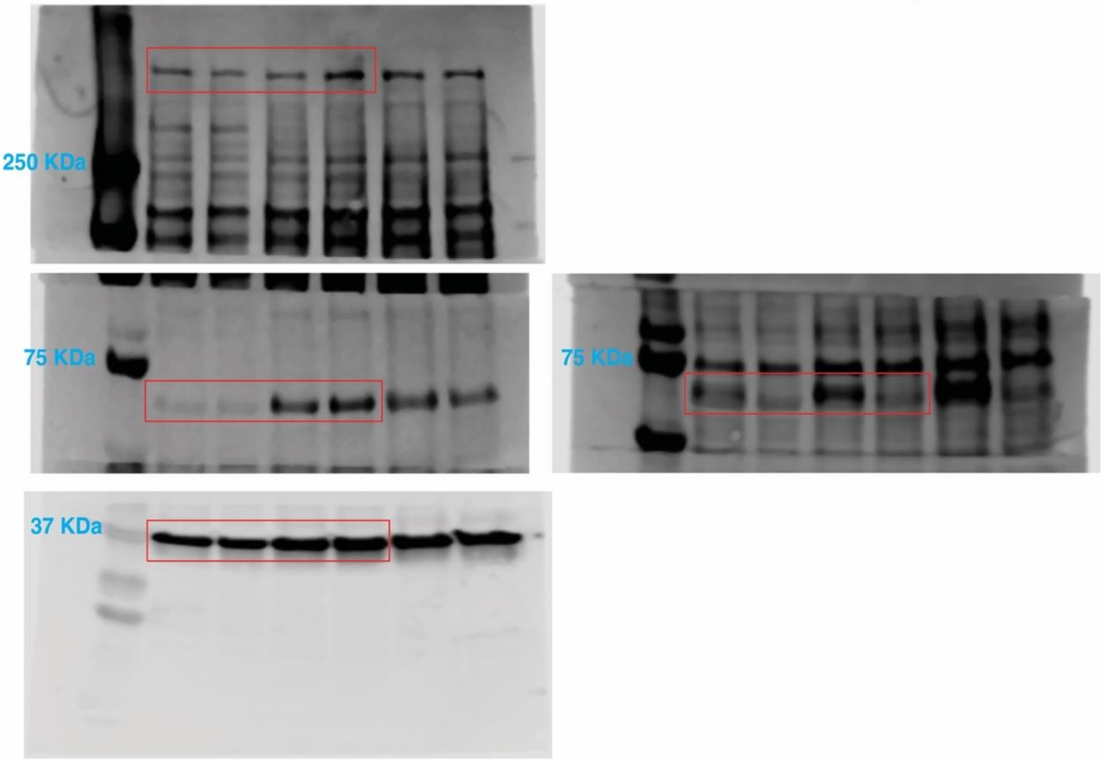

Figure 4E

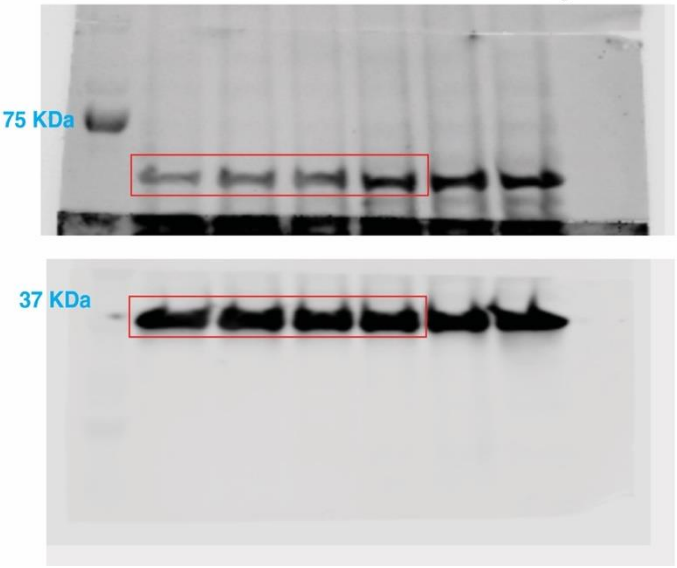

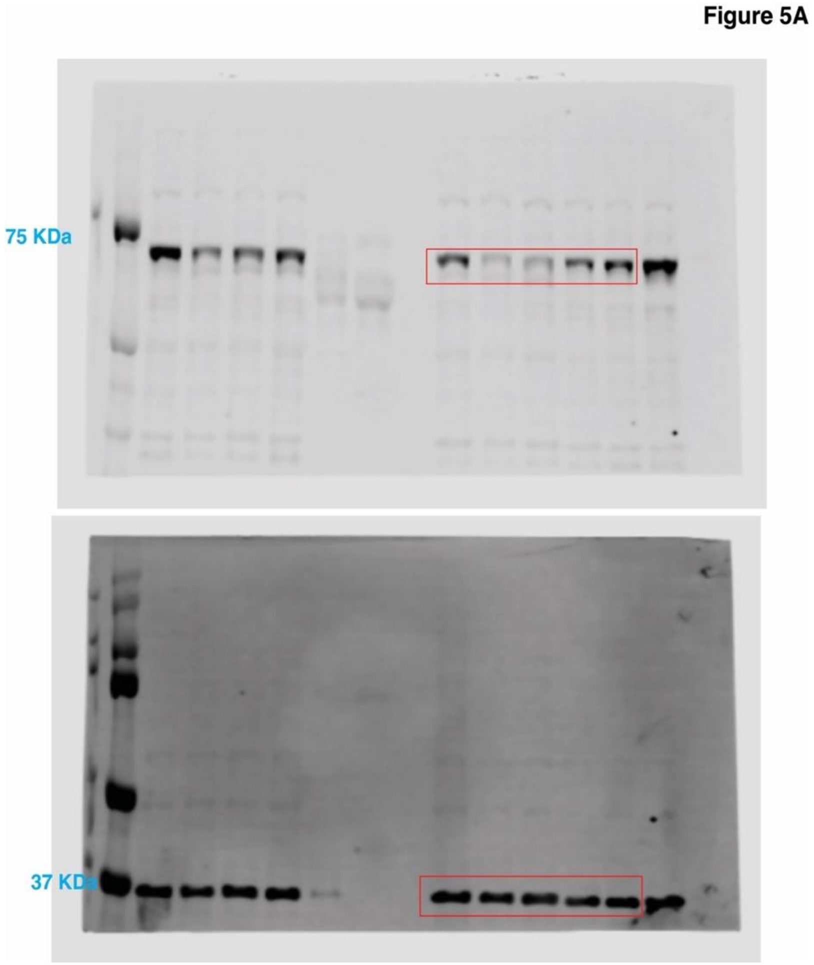
Figure 5A
75 KDa
37 KDa

Supplementary Figure 1B

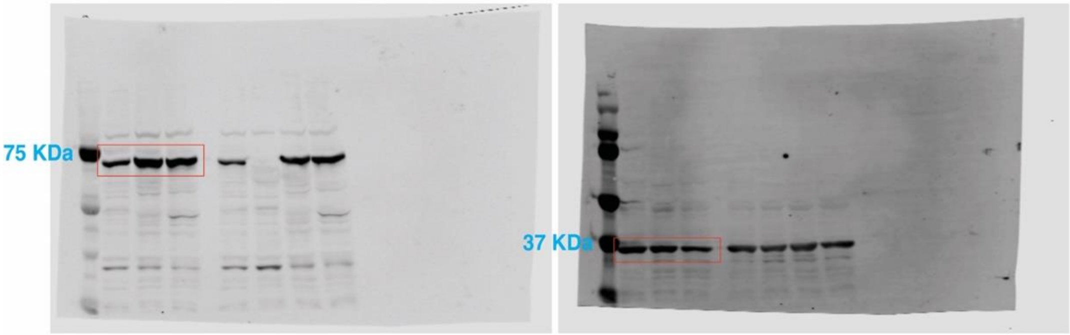

Supplementary Figure 2C

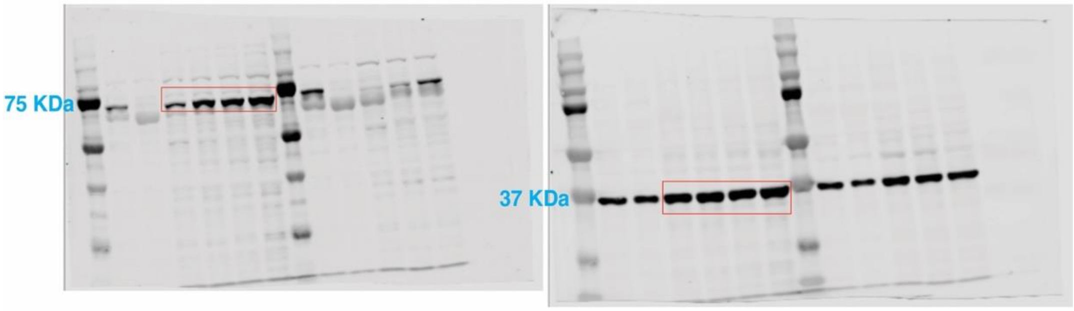

Supplementary Figure 2D

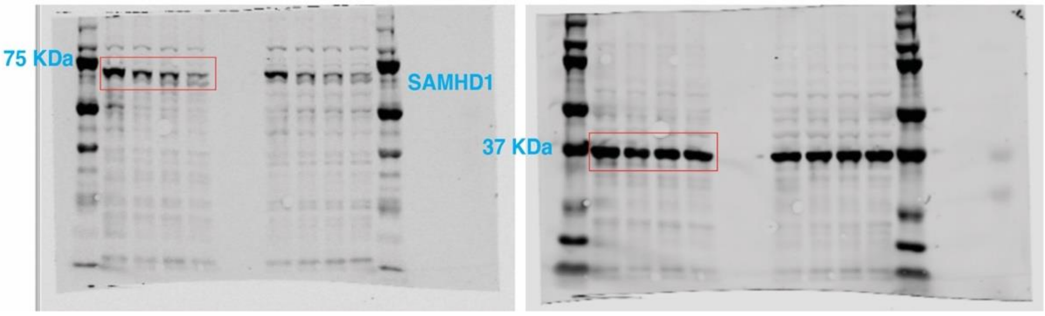

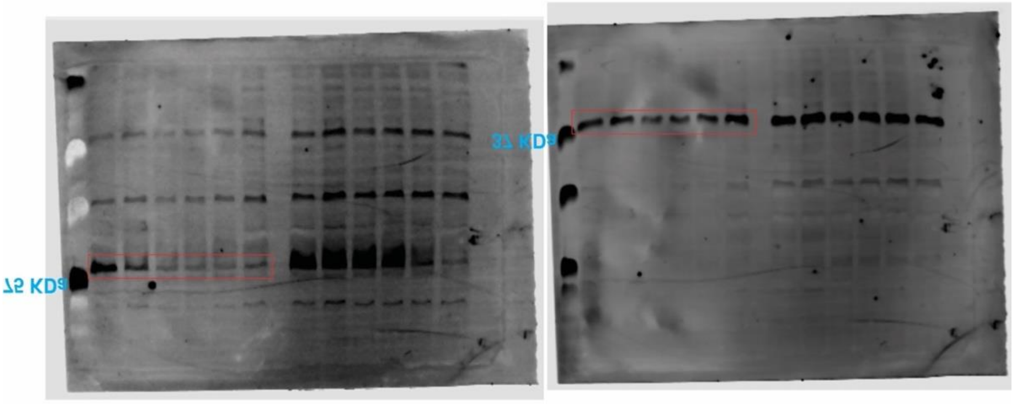

Supplementary Figure 3B

Supplementary Figure 3G

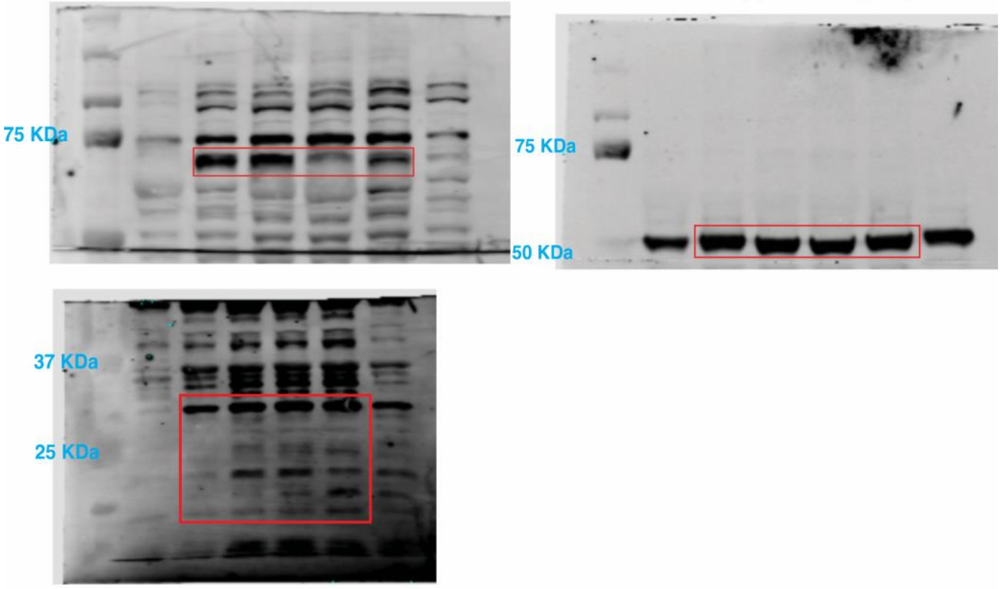

Supplementary Figure 4

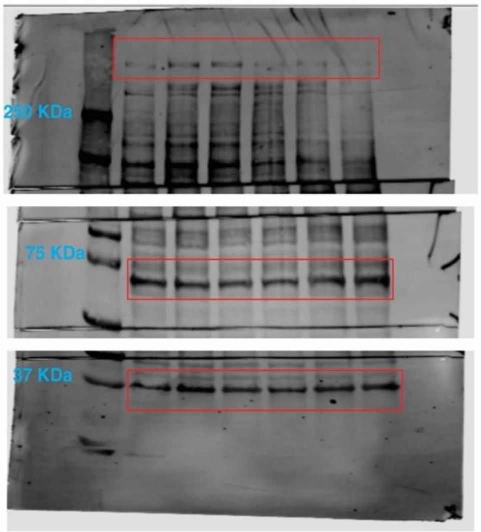

Supplement: Supplementary file 1 [file cancers-14-04490-s001.zip › cancers-1899535-supplementary.pdf]
